# Stepping Into the Breach of Federal Inaction: Reforming the Financing of Long-Term Services and Supports

**DOI:** 10.1093/geroni/igaa057.2528

**Published:** 2020-12-16

**Authors:** Marc Cohen, Edward Miller, Judith Feder

## Abstract

The growing need for long-term services and supports (LTSS) poses significant challenges to both individuals and government. For the current cohort of adults aged 65 years and over, it is estimated that the total cost of LTSS (including the value of care provided by family members) will exceed $6.3 trillion. These staggering national costs are driven by the large and rapidly growing number of individuals in need, their longer life expectancies, significant caregiver shortages, and the high costs of care. Neither private health insurance nor Medicare cover these expenses; Medicaid serves as a safety net for individuals once they have impoverished themselves. With limited to no retirement savings, most older adults cannot cover this liability. Moreover, few people have private long-term care insurance to cover this risk and the private market for such insurance is troubled. Thus, the ability to pay for LTSS is sharply bifurcated: people must be either be rich enough to pay out-of-pocket, or poor enough to qualify for Medicaid. However, even Medicaid-eligible individuals may not be able access needed care due to the waiting lists common in Medicaid. The purpose of this symposium is to document the continuing failure to tackle this problem by the federal government, and to review state and national proposals and initiatives aimed at filling the void left by federal inaction. Marc Cohen and Edward Miller will serve as presenters and chair and co-chair, respectively; Eileen Tell will also present; Judy Feder will serve as the discussant.

